# Rapid multi-criterial design of microwave components with robustness analysis by means of knowledge-based surrogates

**DOI:** 10.1038/s41598-023-32816-w

**Published:** 2023-04-12

**Authors:** Slawomir Koziel, Anna Pietrenko-Dabrowska

**Affiliations:** 1 Engineering Optimization and Modeling Center, Reykjavik University, 102 Reykjavik, Iceland; 2 Faculty of Electronics, Telecommunications and Informatics, Gdansk University of Technology, 80-233 Gdansk, Poland

**Keywords:** Electrical and electronic engineering, Computational science

## Abstract

Manufacturing tolerances and uncertainties concerning material parameters, e.g., operating conditions or substrate permittivity are detrimental to characteristics of microwave components. The knowledge of relations between acceptable parameter deviations (not leading to violation of design specifications) and the nominal performance (not considering uncertainties), and is therefore indispensable. This paper proposes a multi-objective optimization technique of microwave components with tolerance analysis. The goal is to identify a set of trade-off designs: nominal performance versus robustness (quantified by the maximum input tolerance values that allow for achieving 100-percent fabrication yield). Our approach exploits knowledge-driven regression predictors rendered using characteristic points (features) of the component’s response for a rapid evaluation of statistical performance figures, along with trust-region algorithm to enable low execution cost as well as convergence. The proposed methodology is verified with the use of three microstrip circuits, a broadband filter, and two branch-line couplers (a single- and a dual-band one). It is demonstrated that a Pareto set w.r.t. nominal performance and robustness objectives can be produced using only 40 to 60 EM simulations of the respective structure (per design). Reliability of the proposed algorithm is corroborated with the use of EM-based Monte Carlo simulation.

## Introduction

Inherently imperfect manufacturing procedures are the primary sources of uncertainties for microwave designs, including planar and multi-layer structures as well as waveguide components. Deviations of circuit dimensions from the nominal values may cause a potential violation of performance specifications. The latter are becoming continuously stringent, either due to demands implied by emerging application areas (5G communications^1^, internet of things^2^, wearable/implantable devices^3^), functionality requirements (wideband^4^, multi-band operation^5^, reconfigurability^6^), or miniaturization trends^7^. Their fulfilment is challenging even without accounting for tolerances, therefore, only a little margin is normally available to accommodate both statistical deviations and other types of uncertainties, including incomplete knowledge of material parameters, or changing environmental conditions such as temperature, mechanical deformations, etc. Needless to say, the capability of accurate assessment of the tolerance effects is of fundamental importance for ensuring design robustness. It should also be emphasized that improving the nominal performance (e.g., minimization of in-band return loss, maximization of bandwidth, etc.) generally leads to different parameter setups than those corresponding to the most robust designs^8,9^. Consequently, designs of the highest practical utility are frequently sub-optimal with respect to nominal performance figures while ensuring broad enough safety margins for accommodating the tolerances.

Having the circuit topology established, the improvement of the (nominal) performance of passive microwave components is accomplished by appropriate adjustment of geometry parameters. For best results, both in terms of concurrent adjustment of system variables but also handling multiple objectives and constraints, parameter tuning should be realized using numerical optimization techniques^10–12^. For reliability, the computational model of choice is usually based on full-wave electromagnetic (EM) analysis. As the optimization process requires repetitive simulations of the circuit at hand, the associated computational expenses may be high even for local (e.g., gradient-based) algorithms^13^, let alone global search procedures^14,15^. The latter are typically nature-inspired population-based methods^16–19^, the cost of which is measured in thousands of circuit analyses. Incorporating the effects of fabrication tolerances into the design process, in particular, when seeking performance-robustness trade-offs, makes the design task a multi-objective one^20^. In computational terms, multi-objective optimization (MO) poses a more challenging task than single-objective design, and direct EM-driven MO is prohibitively expensive in most practical situations. A common workaround is surrogate-assisted optimization^21,22^. The metamodels, often constructed in an iterative manner during the optimization run^23^, are utilized as fast predictors, with EM analysis employed for verification purposes and model refinement^24^. The modelling techniques popular in this context include approximation ones (kriging^25^, Gaussian process regression, GPR^26^, support-vector machines^27^, neural networks^28^, performance-driven surrogates^29,30^) as well as physics-based methods (space mapping^31^, Pareto-ranking-based bisection^32^, sequential domain patching, SDP^33^).

Evaluating the robustness of microwave designs with respect to fabrication tolerances requires statistical analysis^34^. Quantification of uncertainties involves a definition of a suitable statistical figure of merit, which for microwave components is often the yield^35^. The reason is that design specifications are typically formulated in a minimax sense^36^. Consequently, robust design procedures would aim at yield maximization^37,38^, which is equivalent to improving the probability of having the design requirements fulfilled with the assumed input tolerance levels. These levels are expressed as probability distributions pertinent to the manufacturing process but also possible correlations between the system variables. Robust design problem can also be formulated in terms of finding the maximum parameter deviations for which the design specifications are still satisfied (e.g., design for maximum input tolerance hypervolume, MITH^39^). Regardless of the problem statement, uncertainty quantification (UQ) is CPU intensive when executed with the use of traditional methods such as EM-based Monte Carlo (MC) analysis. Practical UQ procedures largely incorporate surrogate modeling techniques^40–42^. Among these, polynomial chaos expansion (PCE)^43^ has recently become particularly popular in high-frequency electronics^44–47^ because it directly estimates statistical moments (mean, variance) of the circuit responses without the necessity of executing MC.

A limited number of frameworks for multi-objective design of high-frequency components (primarily antennas) and tolerance analysis have been reported. MO of antenna structures and arrays, in which MITH is estimated by means of machine learning methods involving GPR surrogates has been proposed in Ref.^39^. In Ref.^48^, the authors employed kriging interpolation metamodels for robust multi-objective design of high-frequency components with worst-case analysis carried out based on trade-off designs generated by nature-inspired algorithms (here, PSO^49^). Explicit handling of input tolerance hypervolume has been presented in^50^, with the use of non-dominated sorting genetic algorithm (NSGA-II)^51^. The optimization process has been accelerated by means of an ensemble of competing surrogates (polynomial regression, GPR, kriging).

The subject of this work is a novel algorithmic approach to multi-criterial optimization of passive microwave circuits with explicit robustness improvement. Our methodology handles the tolerance-related figure of merit as one of the design objectives. Here, we define it as the maximum value of design variables’ deviations which ensures design specification fulfilment, i.e., yield equal to 100-percent. The second objective is related to the nominal performance of the circuit (e.g., in-band return loss level for filtering structures, power split ratio for the operating frequency for coupling circuits, etc.). Uncertainty quantification is realized using (linear) knowledge-based predictors established using characteristic points (response features) derived from EM-simulated circuit characteristics. Identification of the Pareto-optimal design with regard to the robustness- and performance-related goals is carried out sequentially, by local adjustment of the circuit dimensions for several target values of (nominal) figures of merit. Our procedure is demonstrated using a broadband bandpass filter and two equal-split branch-line couplers. In all cases, the average computational overhead of the MO process is around fifty EM simulations per trade-off design. Reliability of the procedure is corroborated using EM-based Monte Carlo simulation at chosen Pareto-optimal points. The original components of our approach encompass: (1) the development of a multi-criterial design framework with explicit handling of the circuit robustness, (2) the employment of fast response feature predictors, constructed using the knowledge of the system under design, enabling rapid and accurate uncertainty quantification, (3) demonstration of the procedure efficacy when solving real-world tolerance-aware MO tasks in multi-dimensional parameter spaces. The presented methodology can be applied for cost-efficient generation of alternative designs constituting achievable trade-offs between robustness and nominal figures of merit, facilitate decision making processes (e.g., concerning a selection of the manufacturing procedure), as well as meaningful comparison of different circuit topologies from the point of view of their immunity to fabrication tolerances.

### Multi-criterial tuning of microwave circuits with explicit robustness handling

This section introduces the proposed framework for multi-criterial tuning of microwave circuits with explicit handling of fabrication tolerances. We aim at identifying a set of designs ensuring trade-off between robustness versus nominal performance of the circuit. Towards this end, “MO with tolerance analysis: Problem formulation” 1 provides a rigorous formulation of the design task. The details of uncertainty quantification procedure involving knowledge-based response feature predictors are given in “Uncertainty quantification procedure”. “Generating Pareto front” discusses iterative generation of the trade-off points, whereas “Multi-criterial optimization procedure” epitomizes the operation of the overall optimization framework.

## MO with tolerance analysis: problem formulation

We start by introducing the necessary notation. Let ***x*** = [*x*_1_ … *x*_*n*_]^*T*^ stand for a vector of independent (adjustable) parameters of the circuit under design. We assume that the circuit response is simulated using full-wave EM analysis. Typically, we are interested in S-parameters versus frequency, which will be represented by *S*_*ij*_(***x***,*f*) (e.g., *i* =* j* = 1 for reflection characteristic at Port 1, etc.), where f stands for frequency.

### Performance-related objective

Here, multi-objective design is carried out according to two objectives, the performance- and robustness-related ones. A merit function quantifying the nominal performance of a circuit, that is, without parameter deviations arising from fabrication tolerances (or, in general, different types of uncertainties), will be denoted as *F*_*p*_(***x***). We start by presenting two examples.

#### Example 1: bandpass filter

Let us describe a simplified setup (for the sake of clarity) with the only specifications pertaining to the reflection characteristics. Denoting by *f*_*L*_ and *f*_*R*_ the frequencies delimiting the target bandwidth, and *S*_max_ the maximal permitted in-band reflection value, the condition for satisfying the specs at the vector ***x*** can be defined as

1 $$\max \left\{ {f \in \left[ {f_{L} ,f_{R} } \right]\;:\;|S_{11} ({\varvec{x}},f)|} \right\} \le S_{\max }$$

Observe that additional requirements may be imposed in practice, simultaneously on the reflection and transmission characteristics (e.g., the highest level of in-band ripple^52^, maximum out-of-band transmission, etc.).

#### Example 2: multi-band coupler

Let *f*_*R.k*_ and *f*_*L.k*_ be the upper and lower frequencies determining the *k*th bandwidth, *k* = 1, …, *N*, and *D*_*k*_ be the maximal power split error at the *f*_0*.k*_ = 0.5[*f*_*R.k*_ + *f*_*L.k*_]. Further, let *S*_*k*_ denote the intended power split ratio at *f*_0.*k*_. The conditions for satisfying the specs at the design ***x*** are

2 $$\max \left\{ {f \in \bigcup\nolimits_{k = 1}^{N} {\left[ {f_{L.k} ,f_{R.k} } \right]} \;:\;|S_{11} ({\varvec{x}},f)|} \right\} \le S_{\max }$$

3 $$\max \left\{ {f \in \bigcup\nolimits_{k = 1}^{N} {\left[ {f_{L.k} ,f_{R.k} } \right]} \;:\;|S_{41} ({\varvec{x}},f)|} \right\} \le S_{\max }$$

and

4 $$\left| {|S_{31} ({\varvec{{x}}},f_{0.k} )| - |S_{21} ({\varvec{x}},f_{0.k} )| - S_{k} } \right| \le D_{k} \;\;\;\;\;k = 1,...,N$$

The maximum matching/isolation level *S*_max_ is typically set between − 15 and − 20 dB. Having the conditions (2)–(4) satisfied ensures that the circuit exhibits the target operating band (at the level equal to *S*_max_) and, for all operating frequencies, provides the assumed power split (with the required tolerance *D*_*k*_).

Microwave design optimization tasks are frequently formulated in a minimax sense, so that conditions such as (1) or (2)–(4) determine the minimum requirements, whereas the optimization process aims at reducing the levels of relevant responses beyond *S*_max_. For the bandpass filter example considered above, the best nominal design ***x***^*p*^ can be yielded by solving

5 $${\varvec{x}}^{p} = \arg \mathop {\min }\limits_{{\varvec{x}}} \left\{ {\max \left\{ {f \in \left[ {f_{L} ,f_{R} } \right]\;:\;|S_{11} ({\varvec{x}},f)|} \right\}} \right\}$$

For a coupler structure, improving the circuit matching |*S*_11_| and isolation |*S*_41_| (within the assumed operating bandwidths), and maintaining the assumed power split would lead to

6 $${\varvec{x}}^{p} = \arg \mathop {\min }\limits_{{\varvec{x}}} \left\{ {\max \left\{ {f \in \bigcup\nolimits_{k = 1}^{N} {\left[ {f_{L.k} ,f_{R.k} } \right]} \;:\;\max \{ |S_{11} ({\varvec{x}},f)|,|S_{41} ({\varvec{x}},f)|\} } \right\}} \right\}$$

subject to

7 $$|S_{31} ({\varvec{x}},f_{0.k} )| - |S_{21} ({\varvec{x}},f_{0.k} )| = S_{k} \;\;\;\;\;\;\;k = 1,...,N$$

Constraint handling, when solving (6), is usually implicit (using a penalty function approach^53^) because evaluation of (7) is computationally expensive, i.e., requires EM analysis of the circuit.

In either case (here, a filter or a coupler), the nominal performance objective *F*_*p*_(***x***) of our multi-criterial problem will be simply equal to *S*_max_. It should also be mentioned that the performance objective *F*_*p*_ may encapsulate several design goals by itself, e.g., requirements concerning operating bandwidth and power split for the coupler structure considered before. These goals are aggregated to form a single scalar function. Such an approach is convenient because it allows for incorporating the designer’s priorities concerning the objectives. Notwithstanding, a generalized approach is also possible where specific performance-related goals are handled independently. This will be addressed elsewhere.

### Robustness-related objective

We will denote by *F*_*r*_(***x***) a scalar function used to determine the robustness of the system at hand. It is evaluated using a suitable statistical figure of merit, for example, the yield assessed for a given probability density function quantifying the fabrication inaccuracies. In this work, the metric of choice will be the maximum input tolerance levels that ensure meeting the performance specifications (cf. tolerance hypervolume^39^). In practice, the parameter deviations are often modelled using independent normal distributions *N*(0,*σ*) (i.e., with mean equal to zero and variance *σ* common for all variables). If the correlations between the parameter are known (e.g., deviations of the coupled transmission line spacing are highly correlated with the deviation of the line widths), these may be represented using a covariance matrix ***C***, leading to a generic multivariate distribution *N*(0,***C***), which can still be written as *N*(0,*σ****C*’**), where *σ* is a positive scalar.

Observe that the definition *F*_*r*_(***x***) = *σ*(***x***) emphasizes the explicit dependence of *σ* on circuit dimensions. The numerical procedure of evaluating *F*_*r*_(***x***) will be discussed at length in “Uncertainty quantification procedure”.

### MO with tolerance analysis: problem formulation

Having defined the performance- (“Performance-related objective”) and robustness-related objectives (“Robustness-related objective”), we are in a position to formulate the robust multi-criterial design problem as

8 $${\varvec{x}}^{*} = \arg \mathop {\min }\limits_{{\varvec{x}}} \left[ {F_{p} ({\varvec{x}})\;\;\;F_{r} ({\varvec{x}})} \right]$$

The meaning of (8) is to concurrently enhance the intended nominal performance *F*_*p*_(***x***) along with the robust-related objective *F*_*r*_(***x***).

**Figure 1:**
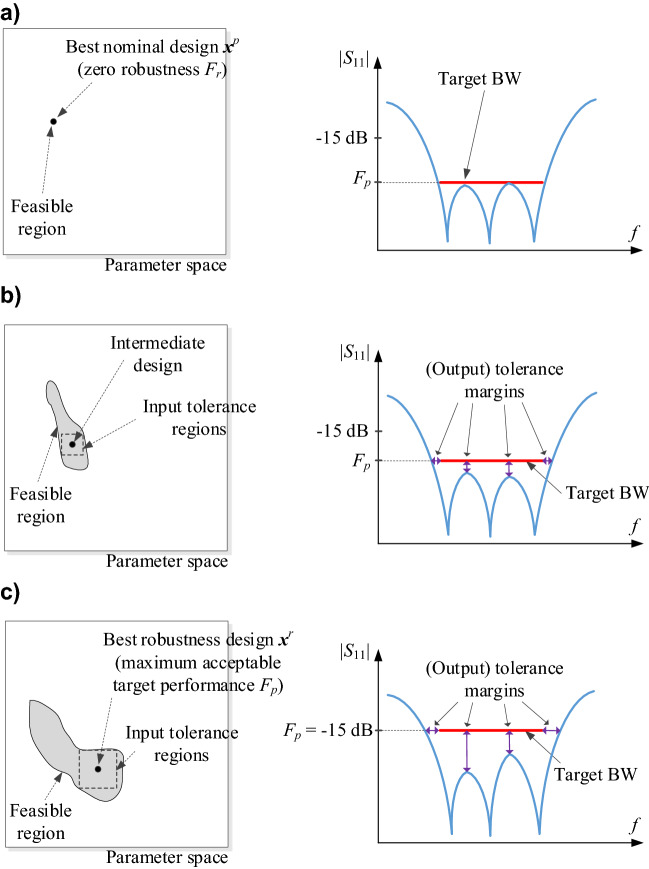
Multi-criterial microwave design with robustness evaluation. Gray manifold indicates the feasible region encompassing the vectors for which, for a given *F*_*p*_, the specifications are met (the feasible region is enlarged for the relaxed *F*_*p*_). The right panels show reflection responses at exemplary designs of a broadband filter for various values of the assumed nominal objective *F*_*p*_: (**a**) the highest-quality nominal design *x*^*p*^; here, the feasible manifold only consists the design *x*^*p*^ itself, consequently, the input tolerance equals 0; (**b**) intermittent design: larger feasible region with the maximum robustness point residing in the centre; (c) the most robust design (for which acceptable value of *F*_*p*_ is the highest, for example, − 15 dB for in-band filter characteristic): the input tolerance values are maximum when centering the design. The design set rendered for various *F*_*p*_ constitute a Pareto set comprising the best robustness vs. performance trade-offs.

It is intuitively clear that the objectives *F*_*p*_ and *F*_*r*_ stay in conflict because stricter performance demands reduce the tolerance for parameter variations. Among available nominal performance versus robustness trade-offs, one can distinguish two extreme situations, namely, the best nominal parameter vector ***x***^*p*^ (cf. “Performance-related objective”), and the design ***x***^*r*^ obtained for the maximum assumed value of *F*_*p*_ that is acceptable for a design task at hand. The latter can also correspond to any other target value according the existing priorities (e.g., − 15 dB maximum in-band reflection level in the case of a filter). Let us briefly characterize these two designs below:

• Vector ***x***^*p*^
  This design realizes the best possible performance with respect to the specifications imposed on the circuit (for example features the minimum reflection within the frequency range of interest). At the same time, ***x***^*p*^ exhibits the lowest possible robustness, especially, the fabrication yield is zero (for any parameter deviation probability distribution); also, the maximum input tolerance levels that ensure fulfilment of the specs are also zero. The reason is that altering ***x***_*p*_ (e.g., by introducing random deviations) necessarily worsens *F*_*p*_(***x***). Further, the design space subset encompassing feasible designs with regard to the performance conditions (e.g., (1) for a filter or (2)–(4) for a coupler) is of the form {***x***^*p*^} (that is assuming a uniqueness of solution to (5) or (6), (7)).
• Vector ***x***^*r*^
  This design is obtained for the most relaxed specification targets, therefore, it is characterized by the largest margin of performance as compared to ***x***^*p*^. This also means that ***x***^*r*^ features the largest robustness as measured by *F*_*r*_. In other words, placing the design in the centre of the feasible region permits to achieve maximum input tolerance levels which, in turn, permit to achieve 100-percent yield. This is because, for maximum *F*_*p*_, the feasible region is of the largest volume.
  The above discussion indicates that designs ***x***^*p*^ and ***x***^*r*^ determining the span of the Pareto front corresponding to the problem (8) are the ‘outermost’ Pareto-optimal points. Multi-objective optimization aims at identifying a discrete group of points being globally nondominated in the Pareto sense^54^ for both considered objectives, *F*_*r*_ and *F*_*p*_. This set will be a representation of the achievable trade-offs between robustness and nominal performance. “Uncertainty quantification procedure”, “Generating Pareto front” and “Multi-criterial optimization procedure” describe the proposed approach to accomplishing this task, whereas Fig. 1 illustrates graphically the discussed concepts.

## Uncertainty quantification procedure

As mentioned in “Robustness-related objective”, the measure *F*_*r*_(***x***) of the circuit robustness is defined to be the highest value of input tolerances, parameterized by the variance σ of the probability distribution selected to describe the parameter deviations’ statistical allocation. The ‘maximum level’ is viewed as the largest value of σ enabling to obtain 100 percent fabrication yield.

### Fabrication yield. Monte Carlo simulation. Robustness objective

The formal definition of yield *Y* at the design ***x*** is^55^

9 $$Y({\varvec{x}}) = \int\limits_{{X_{f} }} {p({\varvec{y}},{\varvec{x}})d{\varvec{y}}}$$

with *p*(***y***, ***x***) being a function of joint probability density that describes the point ***y*** variations with regard to the design ***x***. The feasible space is denoted by *X*_*f*_, i.e., it is the parameter region containing vectors that meet the performance specifications (such as (1) in the case of filters, or (2)–(4) for couplers, cf. “Performance-related objective”). However, the feasible space is not given explicitly, therefore direct integration of (9) is hardly possible. Instead, it is possible to approximate the yield, utilizing, e.g., Monte Carlo (MC) simulation^56^. We have

10 $$Y({\varvec{x}}) = N_{r}^{ - 1} \sum\nolimits_{k = 1}^{{N_{r} }} {H({\varvec{x}}^{(k)} )}$$

The random observables ***x***^(*k*)^ in (10) are of the form ***x***^(*k*)^ = ***x*** + *d****x***^(*k*)^, *k* = 1, …, *N*_*r*_, where *d****x***^(*k*)^ are rendered using the function *p*. Unfortunately, the MC analysis is slowly convergent (the yield estimation speed is proportional to (*N*_*r*_)^−1/2^), so that sizeable datasets are indispensable to provide meaningful results. Consequently, direct EM-driven MC is an expensive procedure. In practice, it is most often expedited using surrogate modeling methods^40–47^.

The definition and the practical ways of estimating the yield are necessary to evaluate the robustness objective. Recall, that *F*_*r*_(***x***) = *σ*(***x***) is the largest variance that ensures 100-percent yield. Consequently, it can be found as

11 $$F_{r} ({\varvec{x}}) = - \arg \mathop {\max }\limits_{\sigma } \left\{ {Y({\varvec{x}},\sigma ) = 1} \right\}$$

The notation *Y*(***x***,*σ*) in (11) is to emphasize that the input probability distribution variance delimits the parameter deviation values, thereby, the yield.

### Response features. Design problem reformulation

For computational-efficiency, in this work, the evaluation of (11) involves knowledge-based predictors rendered using response features as described in the remaining part of this section. Response feature technology serves to expedite and enhance reliability of local optimization of antenna structures^57^. It exploits a close-to-linear dependence between the frequency and level coordinates of adequately chosen characteristic attributes of the circuit responses and the system designable parameters. The feature points are chosen to carry sufficient amount of information from the point of view of whatever design task is to be undertaken, in particular, to determine satisfaction or violation of the assumed performance requirements^57^. Capitalizing on problem-specific knowledge extracted from the system outputs by reformulating design problems in terms of response features brings significant advantages: faster convergence of optimization algorithms^58^, enables global search capabilities even when using local methods^59^, as well as allows for lowering the training data acquisition cost of setting up surrogate models^60^.

**Figure 2:**
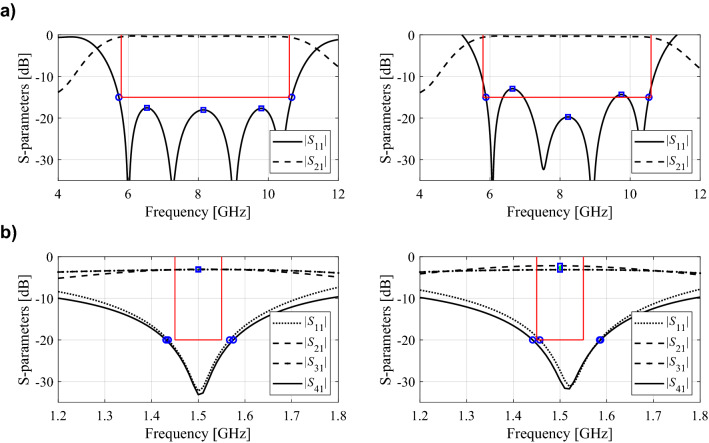
Exemplary features points: (**a**) S-parameters of a bandpass filter, characteristic points refer to − 15 dB level of return loss characteristic along with its local in-band maxima; the features allow for assessing if |*S*_11_| meets the matching conditions within the following band: 5.9 GHz to 10.6 GHz; (**b**) coupler’s S-parameters and the features referring to − 20 dB level of |*S*_11_| and |*S*_41_| characteristics (o), as well as |*S*_21_| and |*S*_31_| at the intended operational frequency 1.5 GHz (◻); the features permit to assess meeting the design requirements within the 100 MHz bandwidth around 1.5 GHz, and maximum power split error equal to 0.5 dB at this frequency. Designs fulfilling and infringing the specs are shown in the left- and right-hand-side panels, respectively.

As mentioned above, the feature point definition depends on design specifications (minimax, *L*-square, frequency-based, level-based) as well as the type of the circuit (filter, power divider, coupler, etc.). If the figure of interest is the circuit operating bandwidth, the feature points would correspond to the frequencies determining the bandwidth at the target level (e.g., − 20 dB) as well as additional points such as local maxima of the return loss characteristics within the frequency range of interest (in the case of filters). Possible allocation of the feature points for two exemplary microwave circuits: a bandpass filter and a coupler can be found in Fig. 2.

Handling response features through numerical procedures requires their rigorous description. Let ***P***(***x***) be a (feature) vector containing the relevant information about the feature points pertinent to a given design task at the vector ***x***. We have

12 $${\varvec{P}}({\varvec{x}}) = [p_{1} ({\varvec{x}})\;p_{2} ({\varvec{x}})\;...\;p_{{N_{p} }} ({\varvec{x}})]^{T}$$

in which *p*_*k*_(***x***) are the coordinates of the feature points (either levels or frequencies). The feature point data is derived from the EM-simulated circuit outputs.

In order to clarify the matter further, let us consider two examples. The first example is a *N*th-order bandpass filter whose design specs are given by (1) (“Performance-related objective”); here, the vector ***P*** has the following form

13 $${\varvec{P}}({\varvec{x}}) = [p_{1} ({\varvec{x}})\;p_{2} ({\varvec{x}})\;...\;p_{N + 1} ({\varvec{x}})]^{T} = [f_{1} ({\varvec{x}})\;f_{2} ({\varvec{x}})\;l_{1} ({\varvec{x}})\;\;...\;\;l_{N - 1} ({\varvec{x}})]^{T}$$

Note that the first two entries are frequencies *f*_1_ and *f*_2_ corresponding to *S*_max_ (such as minus 20 dB) level of |*S*_11_|; the remaining entries *l*_*k*_ refer to in-band maxima of |*S*_11_| (specifically, their reflection levels). The performance requirements (1) may be rewritten in terms of the feature vector as

14 $$f_{1} ({\varvec{x}}) \le f_{L} ,\;\;\;f_{2} ({\varvec{x}}) \ge f_{R}$$

15 $$l_{k} ({\varvec{x}}) \le S_{\max } ,\;k = 1, \ldots ,N{-}{1}$$

Clearly, the filter has to be well tuned to ensure the presence of all maxima; however, this condition holds at the nominal design. In some cases, during optimization run, the number of in-band maxima may be different form that at the nominal design. Nevertheless, such a situation does not occur for the considered parameter variations. In practice, variations being so large that it would affect the shapes of the filter characteristics (and thereby the number of in-band maxima) are unlikely to happen, as this would mean, e.g., error of etching the geometrical details of the filter of large fractions of millimeter (i.e., comparable to their width). The actual manufacturing procedures (chemical etching or mechanical milling) are considerably more accurate with the deviations corresponding to the levels considered in the paper.

Another example is a microwave coupler. If design specifications follow (2)–(4), the feature points should correspond to *S*_max_ (e.g., − 20 dB) levels of |*S*_11_| and |*S*_41_|, but also the values of |*S*_21_| and |*S*_31_| (i.e., coupler’s transmission responses) at the target operating frequency. Therefore, the feature vector is as follows

17 $${\varvec{P}}({\varvec{x}}) = [p_{1} ({\varvec{x}})\;p_{2} ({\varvec{x}})\;...\;p_{6} ({\varvec{x}})]^{T} = [f_{1} ({\varvec{x}})\;f_{2} ({\varvec{x}})\;f_{3} ({\varvec{x}})\;f_{4} ({\varvec{x}})\;l_{1} ({\varvec{x}})\;l_{2} ({\varvec{x}})]^{T}$$

Here, the frequencies of the *S*_max_ level of |*S*_11_| are denoted as *f*_1_ and *f*_2_, whereas the frequencies corresponding to the *S*_max_ level of |*S*_41_| are referred to as *f*_3_ and *f*_4_. Moreover, *l*_1_ and *l*_2_ are the levels of |*S*_21_| and |*S*_31_| at the coupler’s intended operational frequency, respectively (cf. Figure 2b). This concept can be generalized for a multi-band coupler:

18 $$\begin{gathered} {\varvec{P}}({\varvec{x}}) = [p_{1} ({\varvec{x}})\;p_{2} ({\varvec{x}})\;...\;p_{6N} ({\varvec{x}})]^{T} = \\ [f_{1.1} ({\varvec{x}})\;f_{2.1} ({\varvec{x}})\;f_{3.1} ({\varvec{x}})\;f_{4.1} ({\varvec{x}})\;l_{1.1} ({\varvec{x}})\;l_{2.1} ({\varvec{x}})\;\;...\;\;f_{1.N} ({\varvec{x}})\;f_{2.N} ({\varvec{x}})\;f_{3.N} ({\varvec{x}})\;f_{4.N} ({\varvec{x}})\;l_{1.N} ({\varvec{x}})\;l_{2.N} ({\varvec{x}})]^{T} \\ \end{gathered}$$

where the operating band (from 1 to *N*) is indicated by the second subscript.

The design requirements (2)–(4) expressed in terms of the feature points will become

19 $$f_{1.k} ({\varvec{x}}) \le f_{L.k} ,\;\;\;f_{3.k} ({\varvec{x}}) \le f_{L.k} ,\;k = {1}, \ldots ,N$$

20 $$f_{2.k} ({\varvec{x}}) \ge f_{R.k} ,\;\;\;f_{4.k} ({\varvec{x}}) \ge f_{R.k} ,\;k = {1}, \ldots ,N$$

21 $$|l_{1.k} ({\varvec{x}}) - l_{2.k} ({\varvec{x}})| \le D_{k} ,\;k = {1}, \ldots ,N$$

### Knowledge-based predictors

The fundamental benefits of incorporating response feature technology into the uncertainty quantification process is the abovementioned weakly nonlinear (in many cases, close to linear) dependence of the vector ***P*** entries on the circuit dimensions. Consequently, even simple (in particular, linear) metamodels may exhibit predictive power sufficient for reliable statistical analysis in a vicinity of the nominal design. Moreover, the computational cost of setting up such knowledge-based models is considerably lower than for surrogates representing the entire frequency characteristics of the circuit of interest.

Consider a feature-based predictor ***L***_*P*_^(*i*)^(***x***) constructed at the point ***x***^(*i*)^, and representing the vector of response features ***P***(***x***) in the neighborhood of ***x***^(*i*)^

22 $$L_{P}^{(i)} ({\varvec{x}}) = [p_{L.1} ({\varvec{x}})\;...\;p_{{L.N_{p} }} ({\varvec{x}})]^{T} = \left[ \begin{gathered} l_{0.1} + {\varvec{L}}_{1}^{T} ({\varvec{x}} - {\varvec{x}}^{(i)} ) \\ \vdots \\ l_{{0.N_{p} }} + {\varvec{L}}_{{2N_{p} }}^{T} ({\varvec{x}} - {\varvec{x}}^{(i)} ) \\ \end{gathered} \right]$$

Solving linear regression problems ***L***_*P*_^(*i*)^(***x***_*B*_^(*j*)^) = ***P***(***x***_*B*_^(*j*)^) allows for identifying the predictor model, with ***x***_*B*_^(*j*)^, *j* = 1, …, *n* + 1, being trial points, and ***P***(***x***_*B*_^(*j*)^) yielded from EM-simulated circuit response. We have

23 $$\left[ \begin{gathered} l_{0.j} \\ {\varvec{L}}_{j} \\ \end{gathered} \right] = \left[ \begin{gathered} 1\;\;({\varvec{x}}_{B}^{(1)} - {\varvec{x}}^{(i)} )^{T} \\ \vdots \\ 1\;\;({\varvec{x}}_{B}^{(n + 1)} - {\varvec{x}}^{(i)} )^{T} \\ \end{gathered} \right]^{ - 1} \left[ \begin{gathered} p_{j} ({\varvec{x}}_{B}^{(1)} ) \\ \vdots \\ p_{j} ({\varvec{x}}_{B}^{(n + 1)} ) \\ \end{gathered} \right],\;j = {1}, \ldots ,N_{p}$$

Here, the training vectors are allocated by perturbing the center point ***x***_*B*_^(1)^ = ***x***^(*i*)^ as ***x***_*B*_^(*j*)^ = ***x***^(*i*)^ + [0 … 0 *d* 0 … 0]^*T*^, i.e., the (*j−*1)th entry equals *d*. We use the value *d* = 3*σ*, i.e., the perturbations are equal to the largest deviations of the parameters.

### Robustness objective evaluation

Evaluation of the robustness-related objective function (11) requires repetitive computation of the yield, which is realized here by numerically integrating the probability density function *p* in (9). In order to expedite the process, we utilize the feature-based predictor ***L***_*P*_^(*i*)^(***x***), which allows for a fast assessment of design specifications (e.g., conditions (14), (15) for the exemplary filter, or (18)–(20) for the coupler). Estimation of the yield *Y*(***x***,*σ*) is carried out with the use of a large number of random observables ***x***_*r*_^(*j*)^ (in our experiments, we have 100,000 points) rendered following the probability distribution parameterized by the variance *σ*. The routine operates in the following manner:

1. For a variance *σ*, render the set of random observations {***x***_*r*_^(*j*)^}_*j* = 1, …, *Nr*_;
2. For each *j* = 1, …, *N*_*r*_, evaluate the feature-based predictor ***L***_*P*_^(*i*)^(***x***_*r*_^(*j*)^);
3. For all ***x***_*r*_^(*j*)^, *j* = 1, …, *N*_*r*_, verify design specification conditions (e.g., (14), (15), or (18)–(20) using predicted feature coordinates *p*_*L.k*_(***x***_*r*_^(*j*)^);
4. Calculate estimated yield *Y*(***x***,*σ*) as in (10).

Observe that for large *N*_*r*_ the standard deviation of the Monte Carlo process outcome may be greatly reduced. All operations (in particular, Steps 2 and 3) are vectorized to further reduce the CPU cost of yield evaluation.

Having a procedure for fast yield evaluation, the robustness objective *F*_*r*_ is computed by solving (11). Here, as *Y* is dependent on a scalar parameter *σ*, we use a golden ratio search^61^. In general, e.g., joint Gaussian distribution described by a covariance matrix, generic gradient-based procedures may be applied.

## Generating Pareto front

The goal of the proposed multi-criterial optimization with robustness evaluation is to render a discrete sequence of designs being the best attainable robustness versus performance trade-offs. As discussed in “MO with tolerance analysis: Problem formulation”, the Pareto front span is defined by the vector ***x***^*p*^ (the best nominal design, “Performance-related objective”, cf. (5) and (6), (7)), and the vector ***x***^*r*^, representing the best robust design (i.e., that of the maximum acceptable target level *S*_max_).

We aim at generating *N*_*P*_ trade-off designs, starting from ***x***^(1)^ = ***x***^*p*^. Let *S*_max.1_ denote the value of the function *F*_*p*_ at design ***x***^*p*^. For the filter example considered before (cf. (1)), it is

24 $$S_{\max .1} = \max \left\{ {f \in \left[ {f_{L} ,f_{R} } \right]\;:\;|S_{11} ({\varvec{x}}^{p} ,f)|} \right\}$$

For the microwave coupler (cf. (2)–(4)), we have

25 $$S_{\max .1} = \max \left\{ {f \in \bigcup\nolimits_{k = 1}^{N} {\left[ {f_{L.k} ,f_{R.k} } \right]} \;:\max \{ \;|S_{11} ({\varvec{x}}^{p} ,f)|,|S_{41} ({\varvec{x}}^{p} ,f)|\} } \right\}$$

Let us denote as *S*_max.*NP*_ the maximum acceptable level of target *S*_max_. The points ***x***^(*j*)^, *j* = 2, …, *N*_*P*_, i.e., trade-off solutions, are to be rendered for the assumed values *S*_max.*j*_, *j* = 2, …, *N*_*P*_, distributed in equal intervals between *S*_max.1_ and *S*_max.*NP*_ (recall, that *F*_*p*_(***x***^(*j*)^) = *S*_max.*j*_)

26 $$F_{p} ({\varvec{x}}^{(j)} ) = S_{\max .j} = S_{\max .1} + \left[ {S_{{\max .N_{P} }} - S_{\max .1} } \right]\frac{j - 1}{{N_{P} - 1}}$$

Having defined *S*_max.*j*_, the trade-off design ***x***^(*j*)^ is found as

27 $${\varvec{x}}^{(j)} = \arg \mathop {\min }\limits_{{\varvec{x}}} F_{r} ({\varvec{x}})$$

with *S*_max_ set to *S*_max.*j*_ in the relevant objective function, cf. (1) for the filter, and (2)–(4) for the coupler, as well as their response feature counterparts (14), (15), and (18)–(20). In plain words, in order to find ***x***^(*j*)^, the circuit at hand is optimized to improve its robustness as defined by (11), assuming that the target level *S*_max_ = *S*_max.*j*_.

The solution to problem (26) is found iteratively, incorporating the trust-region (TR) principles^62^. More specifically, the design ***x***^(*j*)^ is approximated by a sequence of vectors ***x***^(*j.i*+1)^ produced as

28 $${\varvec{x}}^{(j.i + 1)} = \arg \mathop {\min }\limits_{{||{\varvec{x}} - {\varvec{x}}^{(j.i)} || \le d^{(i)} }} F_{r} ({\varvec{x}})$$

The first of these approximations (i.e., the starting point for the iterative procedure (27)) is the previously obtained trade-off design; we have ***x***^(*j*.0)^ = ***x***^(*j−*1)^. The function *F*_*r*_ (i.e., robustness objective) is evaluated as described in “Robustness objective evaluation”. The solution to (27) is identified in the neighborhood of the current point, given by ||***x ****−**** x***^(*j.i*)^||≤ *d*^(*i*)^, where positive scalar *d*^(*i*)^ constitutes the trust region size parameter; it is re-adjusted upon each consecutive iteration in compliance with the standard TR rules^62^. A decision about accepting or rejecting the new approximate solution ***x***^(*j.i*+1)^ is based upon the gain ratio

29 $$r = \frac{{F_{r}^{\# } ({\varvec{x}}^{(j.i + 1)} ) - F_{r} ({\varvec{x}}^{(j.i)} )}}{{F_{r} ({\varvec{x}}^{(j.i + 1)} ) - F_{r} ({\varvec{x}}^{(j.i)} )}}$$

The denominator thereof quantifies enhancement of the robustness according to the feature-based predictor. The numerator accounts for the actual improvement, although it is still approximated by using an auxiliary function *F*_*r*_^*#*^, calculated as in “Robustness objective evaluation”, still the model ***L***_*P*_^(*j.i*)^ is substituted by ***L***_*P*_^*#*(*j.i*)^, which is built as in (21), (22) yet, instead of the vector [*l*_0.1_ … *l*_0.2*N*_]^*T*^, the feature vector ***P***(***x***^(*j.i*+1)^) derived from EM-simulated response of the circuit at hand at ***x***^(*j.i*+1)^ is utilized. Note that using *F*_*r*_^*#*^ instead of a fully updated surrogate ***L***_*P*_^(*j.i*+1)^ brings tremendous computational advantages because its evaluation only requires one EM analysis. Nevertheless, the assessment made with the use of (28) is generally reliable because the gradients of the features do not change abruptly between subsequent iteration points ***x***^(*j.i*)^ and ***x***^(*j.i*+1)^. The reason is small design relocation (comparable to *σ*) but also weakly nonlinear relation between the coordinates of the features and circuit dimensions.

**Figure 3:**
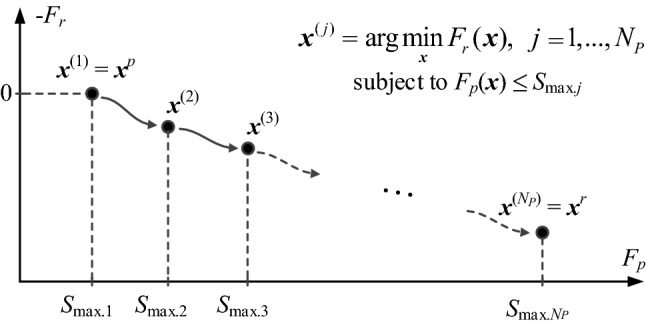
Graphical illustration of multi-criterial design with robustness evaluation. The graph visualizes successive rendition of performance-robustness trade-off solutions, starting from the best nominal design *x*^*p*^.

The design ***x***^(*j.i*+1)^ is approved for positive value of the gain ratio *r*. For *r* < 0, the design is discarded and the iteration is relaunched with a smaller *d*^(*i*)^. On the other hand, if the *r* is close to one (here, *r* > 0.75), the size parameter *d*^(*i*)^ is increased before the next iteration. The algorithm is terminated if either condition is met: (i) ||***x***^(*i*+1)^ − ***x***^(*i*)^||< *ε* (convergence in argument) or (ii) *d*^(*i*)^ < *ε* (shrinking of the trust region). Here, we use *ε* = 10^−3^. The entire multi-criterial optimization procedure has been illustrated in Fig. 3.

### Multi-criterial optimization procedure

**Figure 4:**
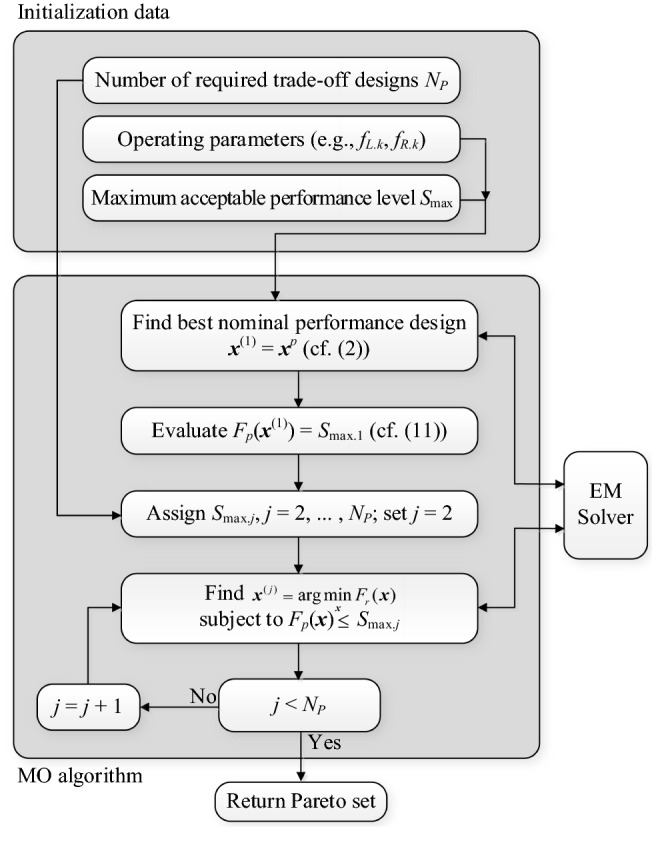
Operating flow of the proposed multi-criterial optimization algorithm with knowledge-based robustness evaluation using feature-based predictors and trust-region-based parameter adjustment.

The operating flow of the proposed multi-criterial optimization algorithm with robustness evaluation has been shown in Fig. 4. The initial setup concerning performance-related design objectives, maximum acceptable performance target level *S*_max_, etc., is up to the user. The best nominal design is usually obtained through a local search. The target performance thresholds *S*_max.*j*_ are determined based on *S*_max_, the performance-related objective value *F*_*p*_(***x***^*p*^), as well as the required number of trade-off points *N*_*P*_. These are identified using the iterative process described in “Generating Pareto front”.

### Verification case studies

This section demonstrates the functioning and performance of the multi-criterial optimization framework introduced in “Multi-criterial tuning of microwave circuits with explicit robustness handling”. Numerical experiments are based on three microstrip structures, a broadband bandpass filter, and two branch-line couplers, a single-band and a dual-band one. The performance-related objective *F*_*p*_(***x***) is formulated using the highest level of reflection characteristic (filter) or matching and isolation characteristics (couplers) within the frequency range of interest. In the latter case, an additional requirement is equal power split condition at the centre frequencies of the respective circuits with 0.5 dB tolerance. The robustness-related performance figure *F*_*r*_(***x***) is given as the maximal variance of the Gaussian distributions characterizing the deviations of the geometry parameters, ensuring the perfect (100 percent) fabrication yield. The obtained results are validated by performing EM-driven Monte Carlo simulation at the chosen trade-off solutions obtained for the considered circuits.

### Case study 1: broadband bandpass filter

**Figure 5:**
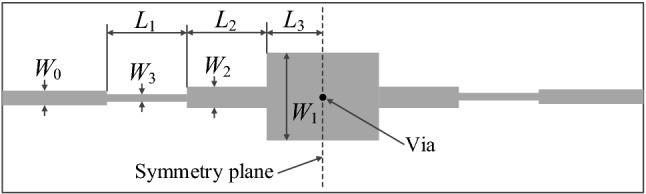
Geometry of the broadband bandpass filter with stepped-impedance resonator^63^.

Consider a broadband bandpass filter with stepped-impedance resonator^63^ presented in Fig. 5. The circuit is implemented on RO4003C substrate (*ε*_*r*_ = 3.55, *h* = 0.305 mm). The design variables are ***x*** = [*L*_1_* L*_2_* L*_3_* W*_1_* W*_2_* W*_3_]^*T*^ (all in millimeters). The width of the feed line equals *W*_0_ = 0.66 mm. The EM simulation model of the filter is evaluated using time-domain solver of CST Microwave Studio.

**Figure 6:**
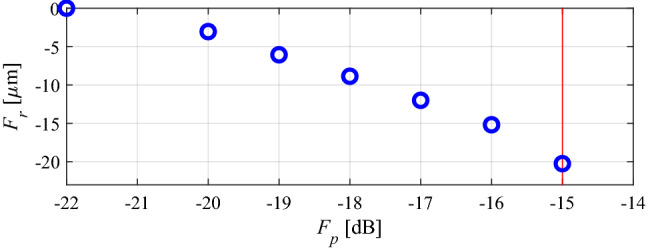
Bandpass filter of Fig. 5: trade-off designs (performance vs. robustness) generated using the multi-criterial optimization procedure of “Multi-criterial tuning of microwave circuits with explicit robustness handling”. The maximal acceptable reflection level within the frequency band of interest is marked using vertical line.

**Figure 7:**
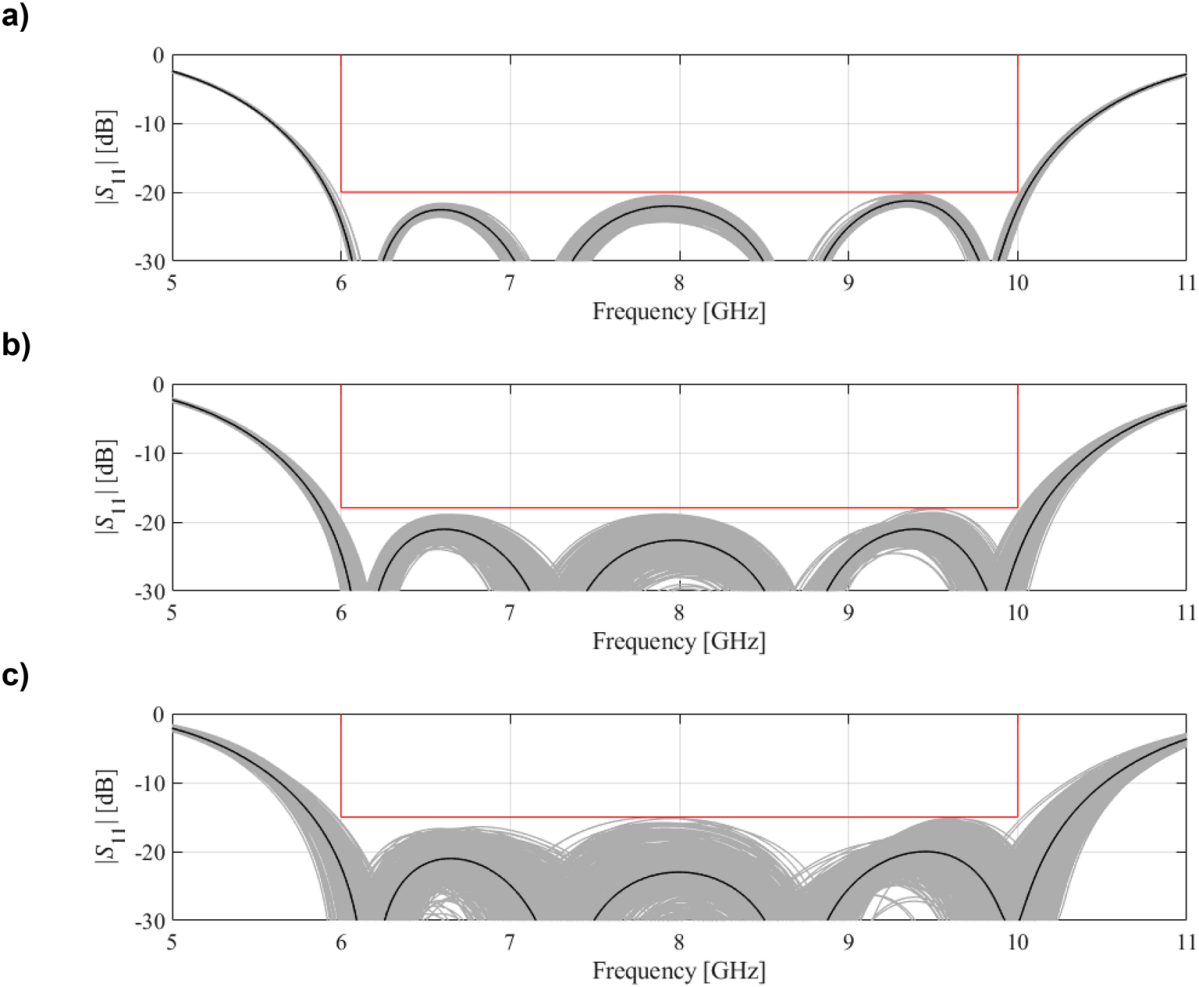
EM-based Monte Carlo analysis of the bandpass filter of Fig. 5 at some trade-off points from Table 1. The circuit output at the selected trade-off design is marked black, whereas 500 random trial points rendered following the probability distribution of the variance σ = *F*_*r*_ are shown using grey color. The specs are indicated by thin lines: (**a**) design *x*^(2)^, (**b**) design *x*^(4)^, (**c**) design *x*^(7)^.

The intended filter operating bandwidth is delimited by *f*_*L*_ = 6.0 GHz and *f*_*R*_ = 10.0 GHz (cf. “Performance-related objective”). The best nominal design *x*^*p*^ = [4.40 5.61 3.76 6.55 1.01 0.48]^*T*^ has been obtained by solving the design task (5), i.e., by minimizing the maximum in-band reflection level |*S*_11_| within the frequency range from *f*_*L*_ = 6 to *f*_*R*_ = 10 GHz. The problem (5) has been solved using the trust-region algorithm, and the achieved maximum in-band reflection is around − 22 dB. At this design, the robustness-related merit function *F*_*r*_(***x***^*p*^) is zero (cf. “MO with tolerance analysis: Problem formulation”). In the course of multi-criterial design, several supplementary trade-off solutions (namely six) have been produced, for *S*_max.2_ = − 20 dB, *S*_max.3_ = − 19 dB, up to *S*_max.7_ = − 15 dB (the highest tolerable value of the reflection). Table 1 provides the relevant data with respect to the trade-off solutions, and Fig. 6 presents the obtained Pareto set. Finally, in Fig. 7, EM-based Monte Carlo (MC) analysis is visualized for some designs of Table 1. MC simulation has been executed for the sake of validation of robustness predictions produced by the feature-based surrogates. More specifically, we need to verify the agreement between the yield maintained during the optimization process (which is supposed to be 100 percent), and its estimation obtained at the level of EM analysis. As it turns out, the MC-based yield is between 99 and 100 percent for all designs ***x***^(2)^ through ***x***^(7)^. At the same time, it should be noted that—due to its high CPU cost—MC was run with only 500 samples, so, the standard deviation of yield estimation is increased.

**Table 1:** Results of multi-criterial design with robustness evaluation for a bandpass filter of Fig. 5

Design	Objectives		Geometry parameters					
	***F***_***p***_** [dB]**	***F***_***r***_** [μm]**	***L***_**1**_	***L***_**2**_	***L***_**3**_	***W***_**1**_	***W***_**2**_	***W***_**3**_
***x***^(1)^ = ***x***^*r*^	− 22	0	4.40	5.61	3.76	6.55	1.01	0.48
***x***^(2)^	− 20	3.1	4.40	5.63	3.77	6.54	1.01	0.48
***x***^(3)^	− 19	6.1	4.39	5.61	3.76	6.54	1.00	0.47
***x***^(4)^	− 18	8.9	4.38	5.60	3.75	6.55	1.00	0.47
***x***^(5)^	− 17	12.0	4.37	5.59	3.74	6.55	1.00	0.47
***x***^(6)^	− 16	15.2	4.36	5.58	3.74	6.54	0.99	0.47
***x***^(7)^ = ***x***^*r*^	− 15	20.3	4.35	5.59	3.73	6.53	0.99	0.47

The proposed multi-criterial optimization procedure is cost-effective. The total cost of rendering the entire Pareto set equals 306 EM simulations of the filter circuit (with the average expenses of generating a trade-off solution corresponding to merely 44 EM analyses). As elaborated on in “Multi-criterial tuning of microwave circuits with explicit robustness handling”, this level of efficiency is mainly possible due to the involvement of the feature-based predictor models.

### Case study 2: miniaturized branch-line coupler

**Figure 8:**
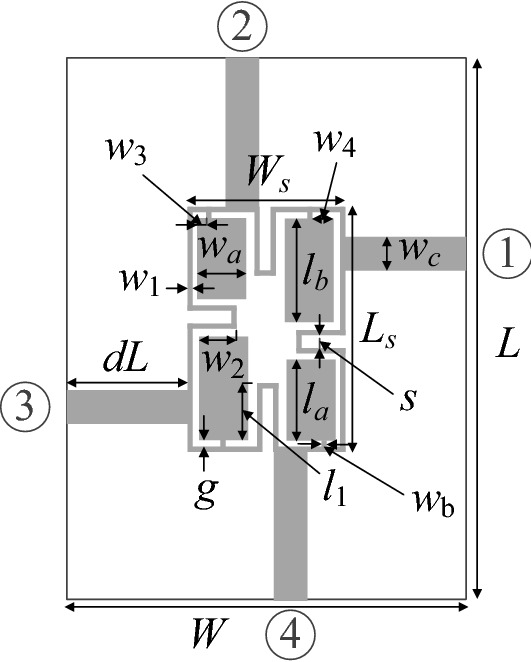
Geometry of the miniaturized microstrip branch-line coupler (BLC)^64^.

Our second verification structure is a compact equal-split branch-line coupler (BLC)^64^ presented in Fig. 8. The circuit is implemented on AD300 substrate (*ε*_*r*_ = 2.97, *h* = 0.76 mm). We have the independent designable parameters: ***x*** = [*g l*_1*r*_* l*_*a*_* l*_*b*_* w*_1_ *w*_2*r*_* w*_3*r*_* w*_4*r*_* w*_*a*_* w*_*b*_]^*T*^ (dimensions in millimeters for absolute parameters; relative parameters indicated by subscript *r* are unitless). Remaining dimensions: *L* = 2*dL* + *L*_*s*_, *L*_*s*_ = 4*w*_1_ + 4* g* + s + *l*_*a*_ + *l*_*b*_, *W* = 2*dL* + *W*_*s*_, *W*_*s*_ = 4*w*_1_ + 4* g* + *s* + 2*w*_*a*_, *l*_1_ = *l*_*b*_*l*_1*r*_, w_2_ = *w*_*a*_*w*_2*r*_, *w*_3_ = *w*_3*r*_*w*_*a*_, and *w*_4_ = *w*_4*r*_*w*_*a*_, *w*_*c*_ = 1.9 mm. The circuit is simulated using transient solver of CST Microwave Studio.

The intended frequency bandwidth is given *f*_*L*.1_ = 1.45 GHz and *f*_*R.*1_ = 1.55 GHz. Furthermore, BLC is to ensure equal power split with the tolerance of 0.5 dB, i.e., the following condition has to be satisfied: ||*S*_21_| −|*S*_31_||≤ *S*_1_ = 0.5 dB at 1.5 GHz. The best nominal performance design ***x***^*p*^ = [0.65 5.93 9.35 12.47 1.29 2.03 0.97 0.32 2.79 0.22]^*T*^ has been obtained by solving the design task (6) and (7) using the TR algorithm with *S*_1_ = 0.5 dB, *f*_*L*.1_ = 1.45 GHz and *f*_*R.*1_ = 1.55 GHz. The nominal design refers to the maximal values of |*S*_11_| and |*S*_41_|, or *F*_*p*_(***x***^*p*^) = − 21.1 dB. For equal-split BLC, six supplementary trade-off solutions were rendered, for *S*_max.2_ = − 20 dB, *S*_max.3_ = − 19 dB, up to *S*_max.7_ = − 15 dB (the highest tolerable level for matching and isolation). Observe that for relative variables, the deviations are re-evaluated to follow the required tolerances of the respective absolute variables taking into account the relationships from the circuit description.

**Figure 9:**
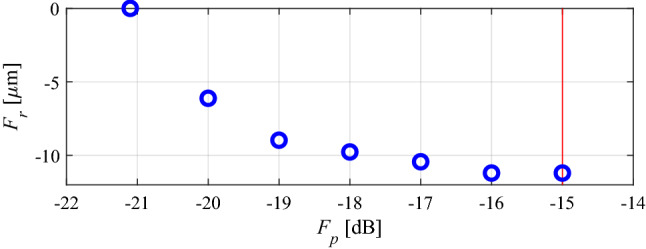
Compact BLC of Fig. 8: trade-off solutions generated using the introduced multi-criterial optimization framework. The maximal acceptable value of in-band |*S*_11_| and |*S*_41_| is marked using vertical line.

**Figure 10:**
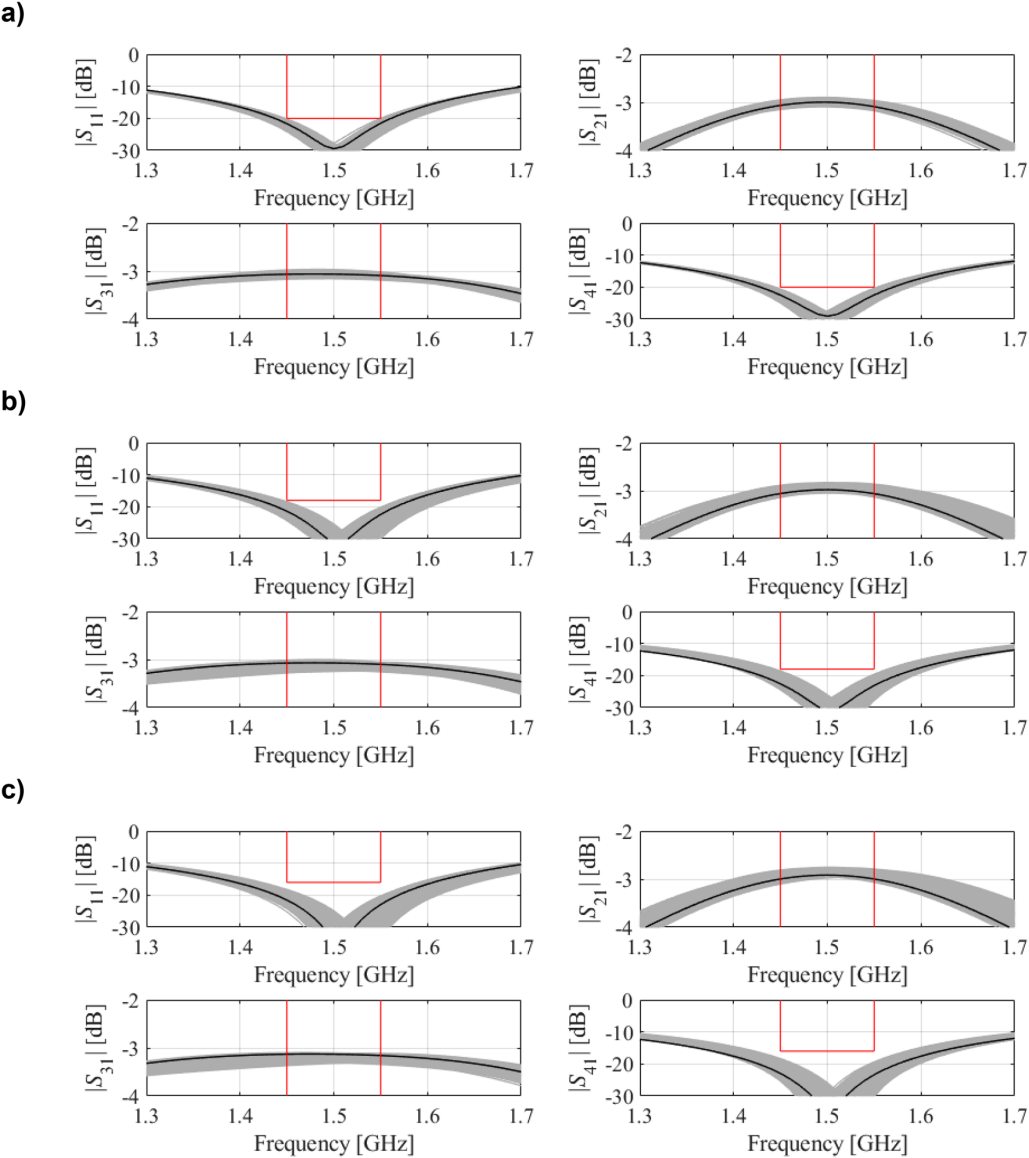
EM-based Monte Carlo analysis of the compact BLC of Fig. 8 at some trade-off points of Table 2. The circuit output at the selected trade-off design is marked black, whereas 500 random trial points rendered following the probability distribution of the variance σ = *F*_*r*_ are shown using grey color. The specs are indicated by thin lines: (**a**) design *x*^(2)^, (**b**) design *x*^(4)^, (**c**) design *x*^(6)^.

Table 2 provides the numerical results, whereas Fig. 9 shows the Pareto front. Figure 10 presents graphical illustration of the EM-driven Monte Carlo analysis. As before, the value of MC-estimated yield nearly equals to 100 percent for all trade-off designs ***x***^(*k*)^. This re-confirms prediction reliability of the feature-based predictors. The total cost of generating the entire Pareto set equals 422 EM simulations of the coupler (with the average expenditures of rendering the trade-off points equal around 60 EM analyses). It should be noted that there is no improvement of the robustness-related objective for *F*_*p*_ higher than − 16 dB, which is due to the fact that the robustness is limited from above by the power split condition (i.e., further relaxing of *F*_*p*_ cannot improve *F*_*r*_ anymore).

**Table 2:** Results of multi-criterial design with robustness evaluation for a compact BLC of Fig. 8

Design	Objectives		Geometry parameters									
	*F*_*p*_ [dB]	*F*_*r*_ [μm]	*g*	*l*_1*r*_	*l*_*a*_	*l*_*b*_	*w*_1_	*w*_2*r*_	*w*_3*r*_	*w*_4*r*_	*w*_*a*_	*w*_*b*_
***x***^(1)^ = ***x***^*r*^	− 21.1	0	0.65	5.93	9.35	12.47	1.29	2.03	0.97	0.32	2.79	0.22
***x***^(2)^	− 20	6.1	0.65	5.93	9.35	12.47	1.29	2.03	0.98	0.33	2.79	0.22
***x***^(3)^	− 19	9.0	0.65	5.91	9.32	12.50	1.32	2.06	1.08	0.37	2.78	0.23
***x***^(4)^	− 18	9.8	0.65	5.95	9.32	12.46	1.32	2.06	1.08	0.39	2.78	0.24
***x***^(5)^	− 17	10.4	0.65	5.93	9.32	12.46	1.32	2.09	1.20	0.44	2.79	0.24
***x***^(6)^	− 16	11.2	0.64	5.94	9.33	12.49	1.32	2.11	1.22	0.44	2.79	0.24
***x***^(7)^ = ***x***^*r*^	− 15	11.2	0.64	5.94	9.33	12.49	1.32	2.11	1.22	0.44	2.79	0.24

### Case study 3: dual-band microstrip branch-line coupler

**Figure 11:**
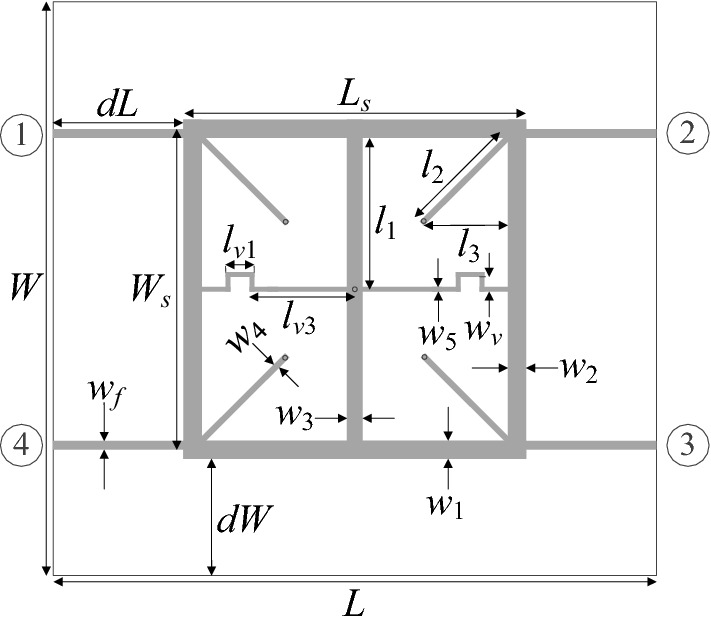
Geometry of dual-band branch-line coupler^65^.

Our last verification structure is a dual-band branch-line coupler (BLC)^65^ of Fig. 11. The circuit is implemented on RO4003 substrate (*ε*_*r*_ = 3.5, *h* = 0.51 mm). The independent design parameters are ***x*** = [*L*_*s*_* W*_*s*_* l*_3*r*_* w*_1_* w*_2_* w*_3_* w*_4_* w*_5_* w*_*v*_]^*T*^. Absolute dimensions are expressed in mm. The parameter *l*_3*r*_ is relative and unitless. Further, we have: *d*_*L*_ = *d*_*W*_ = 10 mm, *L* = 2*d*_*L*_ + *L*_*s*_, *W* = 2*d*_*W*_ + 2*w*_1_ + (*W*_*s* _− 2*w*_*f*_), *l*_1_ = *W*_*s*_/2, *l*_2_ = *l*_3_2^1/2^, *l*_3_ = *l*_3*r*_((*L*_*s*_ − *w*_3_)/2 − *w*_4_/2^1/2^), *l*_*v*1_ = *l*_3_/3, *l*_*v*3_ = *L*_*s*_/2 − *w*_3_/2 − *l*_3_ + *l*_*v*1_; *w*_*f*_ = 1.15 mm. The BLC is simulated using CST Microwave Studio. In the case of the relative variable, the corresponding parameter deviations are recalculated to match the tolerance of the respective absolute variable, here, *l*_3_ (using the dependencies provided above).

The target operating bandwidths are given by *f*_*L*.1_ = 2.35 GHz and *f*_*R*.1_ = 2.45 GHz (lower band), and *f*_*L*.2_ = 5.15 GHz and *f*_*R*.2_ = 5.25 GHz (upper band). Furthermore, the BLC is to ensure equal power split with the tolerance of 0.5 dB, i.e., the following condition has to be satisfied: | |*S*_21_| −|*S*_31_| |≤ *S*_1_ = *S*_2_ = 0.5 dB at the operational frequencies equal to 2.4 GHz and 5.2 GHz. The best nominal performance design ***x***^*p*^ = [25.03 0.80 0.78 1.96 1.29 0.41 0.79 0.34 0.34]^*T*^ (corresponding to the maximum reflection *F*_*p*_(***x***^*p*^) = − 21.5 dB) has been obtained by solving the design task (6) and (7) using the TR algorithm with *S*_1_ = *S*_2_ = 0.5 dB, *f*_*L*.1_ = 2.35 GHz and also *f*_*R.*1_ = 2.45 GHz, *f*_*L*.2_ = 5.15 GHz and *f*_*R.*2_ = 5.25 GHz. Similarly as for the previous example, six supplementary trade-off points have been rendered, for *S*_max.2_ = − 20 dB, *S*_max.3_ = − 19 dB, up to *S*_max.7_ = − 15 dB.

**Figure 12:**
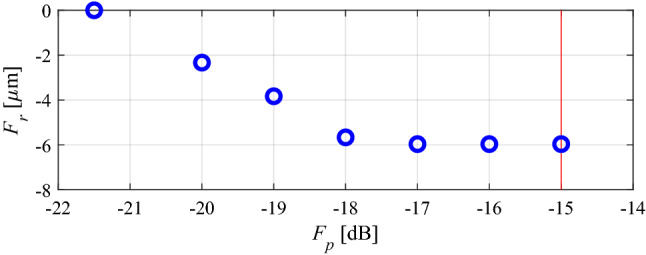
Dual-band BLC of Fig. 11: trade-off designs rendered using the introduced multi-criterial optimization framework. The maximal acceptable value of in-band |*S*_11_| and |*S*_41_| is marked using vertical line.

**Figure 13:**
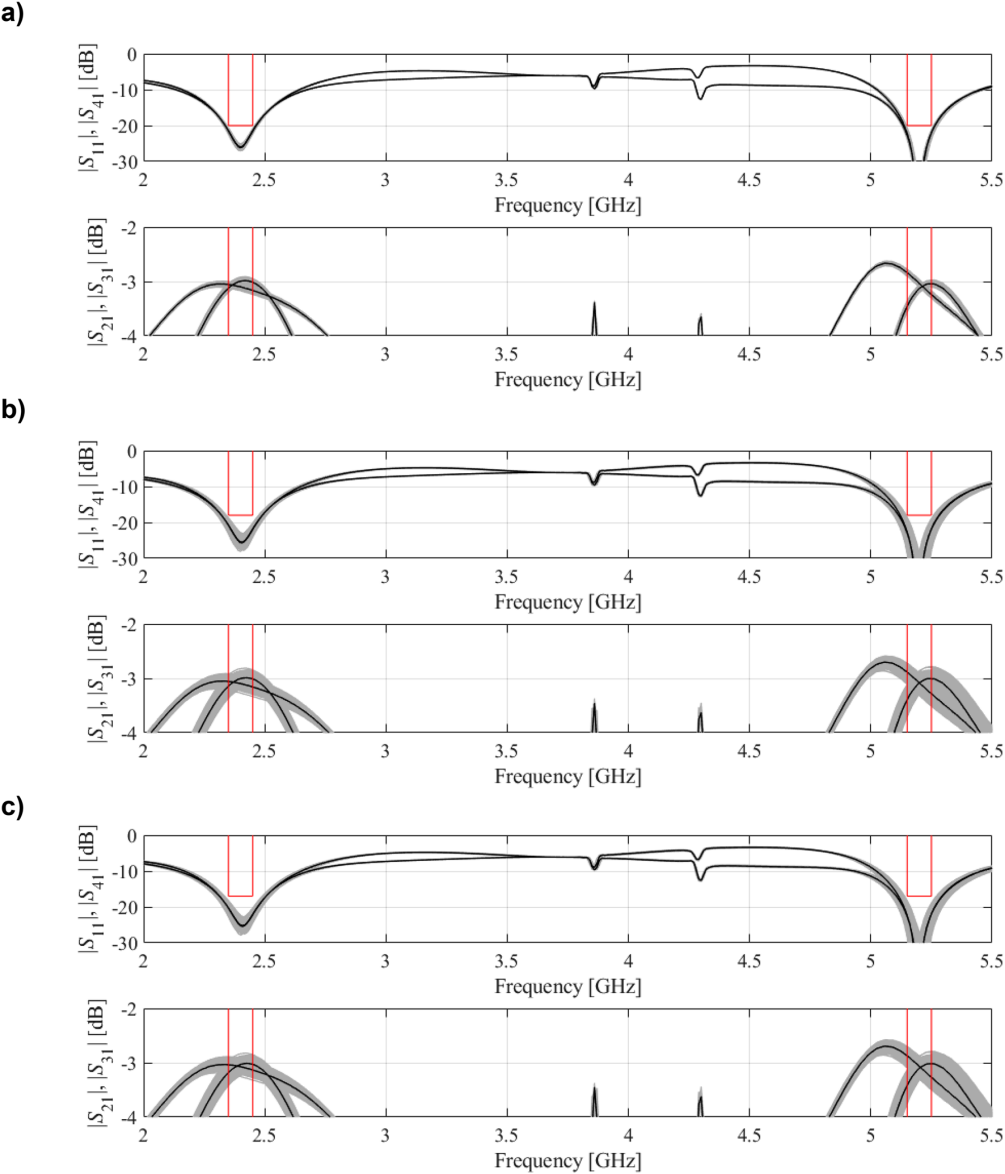
EM-based Monte Carlo analysis of the dual-band BLC of Fig. 11 at some trade-off points of Table 3. The circuit output at the selected trade-off design is marked black, whereas 500 random trial points rendered following the probability distribution of the variance σ = *F*_*r*_ are shown using gray color. The specs are indicated by thin lines: (**a**) design ***x***^(2)^, (**b**) design ***x***^(4)^, (**c**) design ***x***^(5)^.

Table 3 gathers numerical results, and Fig. 12 gives the Pareto set. Whereas Fig. 13 presents results of the simulation-based MC analysis. MC-evaluated yield, for all trade-off solutions, is around one hundred percent. The total cost of generating the entire Pareto set equals 341 EM simulations of the coupler, which corresponds to just 49 EM simulations of the BLC per design, which is consistent with the previous case studies. Similarly as for the second example, no improvement of the robustness-related objective can be observed beyond a certain value of *F*_*p*_, here, − 17 dB. The reason is the same as for the BLC of Fig. 8: the robustness is limited by the assumed maximum tolerable power split error of 0.5 dB.

**Table 3:** Results of multi-criterial design with robustness evaluation for a dual-band BLC of Fig. 11.

Design	Objectives		Geometry parameters								
	*F*_*p*_ [dB]	*F*_*r*_ [μm]	*L*_*s*_	*W*_*s*_	*l*_3*r*_	*w*_1_	*w*_2_	*w*_3_	*w*_4_	*w*_5_	*w*_*v*_
***x***^(1)^ = ***x***^*r*^	− 21.1	0	25.03	0.80	0.78	1.96	1.29	0.41	0.79	0.34	0.34
***x***^(2)^	− 20	2.3	25.02	0.79	0.78	1.94	1.29	0.38	0.78	0.37	0.34
***x***^(3)^	− 19	3.8	25.02	0.79	0.78	1.94	1.27	0.39	0.78	0.37	0.35
***x***^(4)^	− 18	5.7	25.02	0.81	0.78	1.92	1.27	0.41	0.78	0.37	0.35
***x***^(5)^	− 17	6.0	25.02	0.81	0.78	1.92	1.27	0.43	0.79	0.37	0.35
***x***^(6)^	− 16	6.0	25.02	0.81	0.78	1.92	1.27	0.43	0.79	0.37	0.35
***x***^(7)^ = ***x***^*r*^	− 15	6.0	25.02	0.81	0.78	1.92	1.27	0.43	0.79	0.37	0.35

## Conclusion

This work introduced an algorithmic approach for multi-criterial optimization of microwave components with robustness evaluation. The aim is to render a set of alternate parameter vectors corresponding to the best attainable trade-offs (or Pareto set) in the sense of both the nominal performance of the circuit (i.e., neglecting possible uncertainties, primarily manufacturing tolerances), as well as its robustness. The robustness is understood as the highest possible input tolerance values still ensuring satisfaction of given performance specs with 100-percent likelihood. The proposed procedure capitalizes on rapid uncertainty quantification realized by means of the feature-based predictors, which are demonstrated to be instrumental in ensuring both computational efficiency of the algorithm and the reliability of yield estimation. The iterative rendition of trade-off designs utilizes the trust-region (TR) algorithm, which guarantees process convergence. Verification of the candidate solutions generated in the course of the TR process is carried out by reusing feature point sensitivities, which contributes to additional speedup, at the same time, the process reliability is not degraded. Our methodology has been demonstrated using three microstrip circuits, including a bandpass filter, and two microstrip couplers. The optimization cost varies between 40 and 60 EM analyses of the considered circuit with only slight dependence on the dimensionality of the design space. Accuracy of the feature-based predictors has been confirmed via simulation-driven Monte Carlo analysis of the selected trade-off designs. The MO procedure presented in this paper can be applied in support systems facilitating a selection of fabrication procedures, as well as fast determination of available compromise solutions between electrical performance of the circuit and its fabrication tolerance immunity. Other applications include robustness-related comparisons of various candidate circuit architectures (for a given application area), or determination of the required manufacturing process accuracy as a function of nominal performance demands. In our methodology, only the fabrication tolerances are considered (also referred to as aleatory uncertainties). Handling epistemic uncertainties, for example, the tolerances of dielectric relative permittivity being a result of an incomplete knowledge concerning material parameters, will be the subject of the future work.

## Data Availability

The datasets generated during and/or analysed during the current study are available from the corresponding author on reasonable request. Contact person: anna.dabrowska@pg.edu.pl.
